# Involvement of lysosomal proteins in morphology-driven toxicity of (nano)fibers

**DOI:** 10.1007/s00204-026-04503-y

**Published:** 2026-07-14

**Authors:** Rico Ledwith, Carla Ribalta, Mario Pink, Andrea Haase, Verónica I. Dumit

**Affiliations:** 1 Department of Chemical and Product Safety, German Federal Institute for Risk Assessment (BfR), Berlin, Germany; 2 Institute of Pharmacy, Freie Universität Berlin, Berlin, Germany; 3 Department of Chemical Sciences, University of the Western Cape (UWC), Bellville, South Africa; 4 Unit of Materials and Particulate Hazardous Substances, Federal Institute for Occupational Safety and Health (BAuA), Berlin, Germany

**Keywords:** Frustrated phagocytosis, Carbon nanotubes, Nanofibers, Lysosomal damage, New approach methodologies (NAMs)

## Abstract

Fiber morphology (length > 5 μm; respirable diameter < 3 μm) and biopersistence have been linked to their potential to cause fibrosis, lung cancer, and malignant pleural mesothelioma. Among the mechanisms involved, frustrated phagocytosis occurs when macrophages attempt but fail to fully internalize and clear long rigid fibers. Although nanofibers could meet these criteria and trigger frustrated phagocytosis, their small diameters may enable them to entangle, causing them to lose their fiber-like morphology and affecting their toxicological potential. The toxicological assessment of (nano)fibers relies on animal studies; therefore, there is an urgent need to establish in vitro alternatives. Carbon nanotubes, the most commercially prevalent class of nanofibers, have been extensively investigated, and some have demonstrated pathogenic potential, by causing inflammation initiated by cathepsin B translocation from the lysosomes into the cytosol. Independent studies have indicated that only long and rigid carbon nanofibers lead to a decrease of lysosomal enzymes (including multiple cathepsins) inside macrophages and increased levels in the extracellular environment. Thus, different roles for cathepsin B have been proposed in response to nanofiber exposure. To reconcile these observations, this review examines the underlying mechanisms by assessing in vitro studies, particularly how in vitro macrophages respond to carbon-based nanomaterials of distinct morphologies, discusses the limitations of current in vitro models, and evaluates potential approaches for assessing nanofiber toxicity.

**Supplementary Information:**

The online version contains supplementary material available at https://doi.org/10.1007/s00204-026-04503-y.

## Introduction

Toxicological research on pathogenic fibers, like asbestos, has led to the development of the fiber pathogenicity paradigm (FPP), which connects both material morphology (length and diameter) and biopersistence of respirable fibers to the potential for adverse health effects such as fibrosis, lung cancer, and malignant pleural mesothelioma (MPM) (Pott and Friedrichs 1972; Stanton and Wrench 1972). This paradigm has shaped the World Health Organization’s (WHO) criteria for critical fibers, as those with a length ≥ 5 μm, a respirable diameter ≤ 3 μm and an aspect ratio ≥ 3:1, with the additional criterion of being sufficiently bio-durable (WHO 1997). The accepted mode of action (MoA) underlying fiber toxicity is frustrated phagocytosis, which occurs when alveolar or pleural macrophages attempt, but fail, to engulf long fibers that exceed their size and engulfment capacity (Donaldson et al. 2010; Fortini et al. 2020), estimated for human macrophages to be 10–20 μm (Krombach et al. 1997). Frustrated phagocytosis is a substance-agnostic, morphology-driven process that initiates an inflammatory cascade with recruitment of immune cells, which leads to chronic inflammation, a key factor in cancer development (Boyles et al. 2015; Donaldson et al. 1992; Kopf et al. 2015; Murphy et al. 2011; Nel 2023).

The health hazards associated with inhalation of critical fibers, as revealed by extensive research on asbestos exposure, raise significant concerns, as continuous advances in nanotechnology have led to the widespread presence of nanofibers (NFs) across multiple sectors. Carbon nanotubes (CNTs) are a leading example of NFs, with exceptional properties that can be tailored during synthesis (Nel 2023). They are increasingly used in diverse applications, *inter alia*, energy storage, building composites, heat and electric conductivity, biomedicine, among others (Lee et al. 2022; Maheswaran and Shanmugavel 2022; Rao et al. 2018). Although the morphology of CNTs could fall within the criteria for critical fibers described under the FPP, NFs pose a challenge to the FPP because their small diameters may grant them flexibility, enable them to entangle, and cause them to lose their fiber-like morphology. Consequently, although such entangled CNTs would still have a respirable diameter ≤ 3 μm, they would not fully conform to the FPP (Kane et al. 2018; Nel 2023). A harmonized classification and labelling (CLH) for multi-walled C(N)Ts (MWC(N)Ts) concluded that MWCNTs with diameters below 30 nm tend to form particle-like agglomerates (BAuA 2021; EU Commission 2024), and subsequently their toxicity would align more closely with that observed for bio-persistent granular particles (Lee et al. 2018). In contrast, rigid MWCNTs, such as Mitsui-7, which has a mean diameter of ca. 50 nm and a high proportion of critical fibers (according to the WHO criteria) (Jackson et al. 2015; Porter et al. 2010; Poulsen et al. 2013), has been associated with lung carcinogenicity and MPM in rat studies (Fukushima et al. 2018; Kasai et al. 2016; Numano et al. 2019), leading to its classification as a suspected carcinogen.

Although it appears that the asbestos-like pathogenicity of NFs can be determined by evaluating their rigidity (Kane et al. 2018), their toxicity might additionally be affected by their potential to form secondary structures, such as aligned bundles. In such scenarios, individual pristine NFs with small diameters may be entangled, but secondary structures can conform with the WHO criteria and exhibit fiber pathogenicity (Dumit et al. 2024). This suggests that physicochemical characterization of individual fibers alone may not fully predict the pathogenicity of NFs. Therefore, ensuring the safe use of CNTs, as well as of other NFs, requires comprehensive safety evaluations, which currently still rely on animal testing on a case-by-case basis. Testing strategies must evolve to align with the commitment of the European Commission to the 3Rs principles (replace, reduce, and refine animal use) to phase out animal testing in the area of chemical safety assessment, and to keep up with the rapid emergence of novel NFs. This requires advancing the development of new approach methodologies. However, considering that the long-term effects of NF toxicity include cancer as an adverse outcome, a comprehensive understanding of underlying toxicity mechanisms is fundamental. To this end, Adverse Outcome Pathways (AOPs) provide a valuable framework to describe how a molecular initiating event (MIE) can lead to an adverse outcome through a well-defined sequence of key events (KEs) across molecular, cellular, organ, and organism levels (Tollefsen et al. 2014). Frustrated phagocytosis is the MIE for AOPs addressing fiber toxicity leading to lung cancer (AOP 303) (Seidel et al. 2023) and MPM (AOP 409) (Mansour 2023). However, it is not a well-defined phenomenon that can be measured directly in vitro (Murphy et al. 2021). Although frustrated phagocytosis can be inferred when in vitro macrophages are exposed to pathogenic fibers and are examined microscopically, these methods lack reproducibility, standardization, and high throughput capacity (Mularski et al. 2018). Therefore, in vitro based testing strategies should focus on establishing predictive in vitro biomarkers that are reflective of the MoA of frustrated phagocytosis and subsequent KEs. These biomarkers could help elucidate the morphological characteristics of critical fibers based on their biological response upon exposure to macrophages in vitro (Stobernack et al. 2025).

Notably, the translocation of lysosomal luminal proteins has previously been postulated as such biomarkers. In this context, when devising an assessment strategy for critical fibers from an academic perspective, Murphy et al. (2021) postulated that NLRP3 inflammasome activation is a mechanism involved in macrophages undergoing frustrated phagocytosis. Mechanistically, lysosomal disruption leads to the intracellular translocation of lysosomal luminal proteins, including cathepsin B into the cytosol, which cleaves and activates the NLRP3 inflammasome. Subsequently, the NLRP3 inflammasome mediates caspase-1 activation, which is essential for the maturation and extracellular secretion of the potent pro-inflammatory cytokines interleukin-1β (IL-1β) and IL-18 (Murphy et al. 2021; Wang et al. 2017). However, although increased NLRP3 inflammasome activation has been observed in vivo as a key feature of macrophages undergoing frustrated phagocytosis, and although the increased secretion of proinflammatory mediators is a fundamental KE within AOPs addressing fiber toxicity, these cellular responses are not unique to fiber toxicity and can be activated by various stimuli (Kelley et al. 2019; Lee et al. 2018).

Furthering this investigation, in a previous study conducted by our group, we aimed to elucidate morphology-driven molecular mechanisms and identify unique biomarkers using proteomic techniques. To this end, human-differentiated dTHP-1 macrophages were exposed to Mitsui-7, NM-400 (an entangled MWCNT), and the particulate counterpart, carbon black. Results indicated that only Mitsui-7 caused a dose-dependent decrease in the abundance of lysosomal luminal proteins in the cell lysates, including multiple cathepsins (Ledwith et al. 2025). Subsequent experiments, in which NR8383 rat alveolar macrophages were exposed to NFs, confirmed this effect (Stobernack et al. 2025). Additionally, increased levels of these proteins were detected in extracellular supernatants, providing a mechanistic understanding of the role of these proteins during frustrated phagocytosis (Stobernack et al. 2026). These results, however, do not align with the postulated mechanism by Murphy et al. (2021) for frustrated phagocytosis, where it is accepted that cathepsin B translocates from lysosomes to the cytosol, as this does not explain the decrease of lysosomal proteins in cell lysates, nor their increase in the extracellular supernatant. Consequently, this raises the question of whether there is a unique mechanism driving the extracellular release of lysosomal proteins, and whether this process is linked to the MoA of frustrated phagocytosis, or merely a result of cell death.

The review aims to investigate lysosomal damage and the subsequent translocation of lysosomal luminal proteins, either intracellular or extracellular, as morphology-driven cellular responses to pathogenic NFs, and to explore their role as an in vitro set of biomarkers indicative of frustrated phagocytosis. Selected in vitro studies are reviewed and data from various assays are extracted and summarized across three assessment categories: (i) microscopy evaluations; (ii) intracellular lysosomal enzyme translocation measurements and the associated NLRP3 inflammasome response; and (iii) extracellular lysosomal protein translocation measurements. All endpoints are evaluated alongside cytotoxicity assessment. Unfunctionalized carbon-based nanomaterials (NMs) were selected for this investigation for two main reasons: first, the substantial body of in vitro and in vivo research conducted with these materials; and second, a diameter threshold for rigidity has already been established for this material group in a CLH (BAuA 2021) and included in an adaptation to technical process (EU Commission 2024). This threshold allows for the differentiation and categorization of materials into distinct, morphologically dissimilar groups, enabling the investigation of morphology-driven effects.

## Review of in vitro studies

### Selection criteria for studies considered in this review

**Fig. 1:**
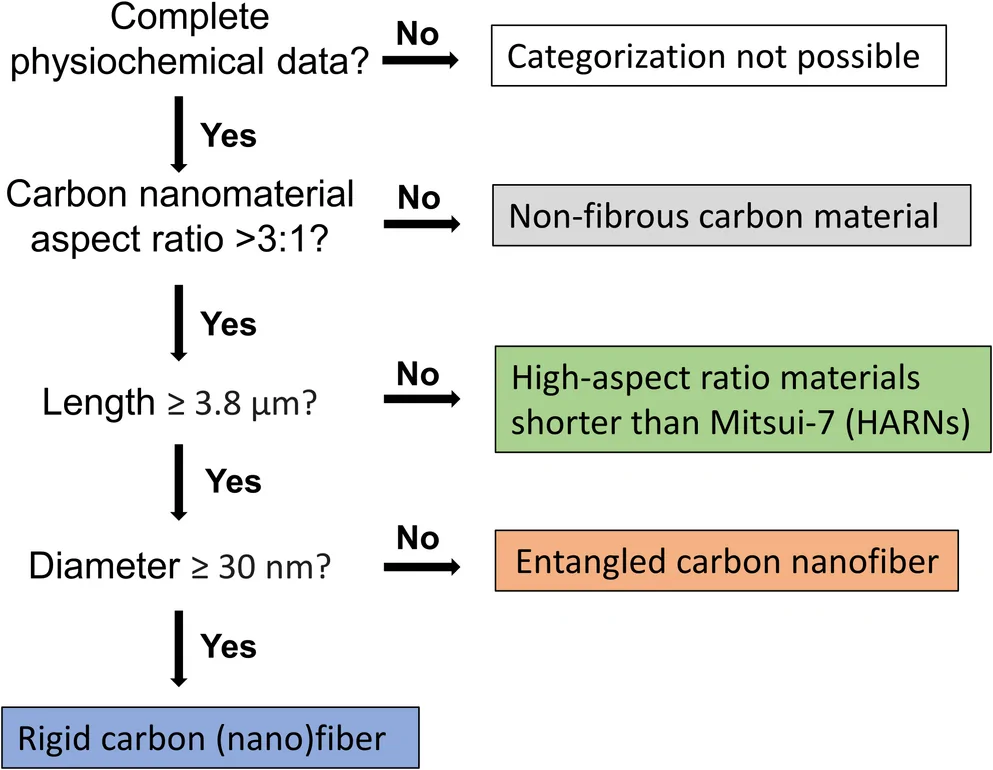
A decision tree categorizing the four groups of carbon-based materials: (1) non-fibrous carbon materials with an aspect ratio ≤ 3:1; (2) high-aspect-ratio NMs with length < 3.8 μm; (3) entangled carbon NFs with length ≥ 3.8 μm and diameter < 30 nm; (4) rigid carbon (nano)fibers with length ≥ 3.8 μm and diameter ≥ 30 nm. Figure adapted from Dumit et al. (2024), licensed under CC BY 4.0 (colour figure online)

Following a literature search, a total of 62 in vitro studies were selected, in which various monoculture macrophage models were exposed to CNTs of various lengths and morphological characteristics, as well as their particulate counterparts. The list of keywords used for the literature search is summarized in Supplementary Table I. The studies are summarized in Supplementary Table II, where the morphological data of the materials, including length and diameter, are used to categorize them into distinct morphological groups, as described in Fig. 1. The reasoning behind this classification was consistency with a meta-analysis that evaluated over 100 omics studies focusing on MWCNT toxicity (Dumit et al. 2024). An aspect ratio of < 3:1 is applied to distinguish between fibrous and non-fibrous materials. The length threshold of Mitsui-7 (average of 3.8 μm) (Poulsen et al. 2013) is used to categorize the exposure material as either a (nano)fiber or a high-aspect ratio material (HARN). Notably, although this length threshold is lower than that outlined by the WHO criteria, it is used because of Mitsui-7’s classification as a suspected carcinogen. It is important to note that studies typically report average lengths, which can conceal the overall size distribution. Fiber length distributions vary and include a fraction of fibers that are longer than the reported average, which may be capable of causing frustrated phagocytosis, as evidenced by the pathogenicity of Mitsui-7 (Fukushima et al. 2018; Kasai et al. 2016; Numano et al. 2019). Lastly, the proxy diameter threshold for rigidity of 30 nm, as established from the CLH proposal, was used to categorize the materials as either rigid or entangled (BAuA 2021). When insufficient physicochemical data for length and diameter are available, categorization into one of the aforementioned groups is not possible, and these materials are not further discussed in this review. Importantly, across all reviewed studies, experimental conditions were limited to exposure durations of ≤ 96 h and exposure concentrations of ≤ 100 µg/mL.

It is worth noting that, when direct visualization of frustrated phagocytosis is intended, the preferred methodology among these studies was cryo-electron microscopy (cryo-EM). This technique enables the observation of morphological changes in cellular structure following phagocytic internalization of the exposed material. It can also reveal alterations in the lysosomal subcellular structure, including alterations in lysosomal size, content disruption, and the localization of the material within the lysosome, including its interaction with the membrane. In contrast, when the goal of the studies is to gain insights into intracellular lysosomal protein translocation, fluorescence microscopy is typically applied. Commonly used methods often include colorimetric assays (e.g., neutral red) to evaluate lysosomal dye retention, and immunoassays or flow cytometry to quantify NLRP3 inflammasome–related markers, including caspase-1, IL-18, and primarily IL-1β. On the other hand, to assess extracellular lysosomal enzyme translocation, the studies usually employed immunoassays, colorimetric and fluorometric activity-based assays, as well as secretome (secretomics) analysis. Overall, these approaches aimed to quantify lysosomal proteins released into cell culture supernatants. The most frequently analyzed enzymes included cathepsins, *N*-acetyl-β-D-glucosaminidase, β-glucuronidase, β-galactosidase, and β-hexosaminidase.

### Microscopy observations

Cryo-EM was employed in 28 out of 62 studies. Various rigid carbon (nano)fibers, including Mitsui-7, were observed to induce frustrated phagocytosis in macrophages, as concluded by the observation of incomplete fiber engulfment, membrane deformation, and (nano)fiber penetration through the macrophage cellular membrane (Boyles et al. 2015; Cheng et al. 2009; Lee et al. 2018; Murphy et al. 2012; Poland et al. 2008; Sanchez et al. 2011). In the case of Mitsui-7, these findings are further supported by in vivo studies, which showed that macrophages isolated from lung fluids exhibited the same signs of frustrated phagocytosis in exposed rodents (Lee et al. 2018; Poland et al. 2008). Similarly, several rigid carbon (nano)fibers were shown to cause lysosomal damage, characterized by the (nano)fiber penetration through the lysosomal membrane into the cytoplasm, often accompanied by lysosomal membrane disruption and lysosomal swelling (Fujita et al. 2020; Keshavan et al. 2021; Svadlakova et al. 2020). In contrast, entangled carbon NFs are effectively phagocytosed and internalized into lysosomes (Kuroda et al. 2018; Murphy et al. 2012), vacuole-like compartments (Cohignac et al. 2018; Fujita et al. 2020) or endocytotic membrane-lined vesicles (Wang et al. 2011). Similarly, HARNs have been visualized to be effectively phagocytosed and compartmentalized into lysosomes (Bussy et al. 2013; Cohignac et al. 2018; Dong et al. 2013, 2020; Gao et al. 2011; He et al. 2018; Keshavan et al. 2021; Long et al. 2019; Murphy et al. 2012; Yang et al. 2014), or vacuole-like compartments (Fujita et al. 2020). Furthermore, all non-fibrous particulate carbon materials have been observed to be compartmentalized in lysosomes (Boyles et al. 2015; Cohignac et al. 2018; Haniu et al. 2011; Murphy et al. 2012; Nishanth et al. 2011; Svadlakova et al. 2020). Collectively, these studies infer that only rigid carbon (nano)fibers, as analyzed by cryo-EM, caused frustrated phagocytosis and lysosomal damage, whereas all other carbon-based NM morphologies, as described in Fig. 1, appeared effectively compartmentalized.

Although Cryo-EM provides evidence that frustrated phagocytosis and lysosomal alterations can be evaluated using this method, the small number of cells captured may not be representative of the overall population. As discussed below, the potential of additional methods is considered for assessing frustrated phagocytosis and lysosomal alterations using biological readouts. Given the substantial evidence that fiber exposure affects lysosomes in macrophages, further investigation using targeted assays to characterize these lysosomal alterations is warranted.

### Intracellular lysosomal enzyme translocation

The intracellular translocation of lysosomal proteins, along with associated NLRP3 inflammasome activation, has been postulated as a hallmark of fiber toxicity. Due to the lysosomal alterations caused by critical (nano)fiber exposure, it is likely that lysosomal proteins beyond cathepsin B are also translocated to the cytosol. Therefore, it is important to examine lysosomal proteins collectively, and their subcellular localization, following fiber exposure. Results are discussed according to the used methodology.

#### Fluorescence microscopy

Qualitative assessments using fluorescence microscopy were employed in nine out of 62 considered studies. These techniques involve the use of lysosomal probes or fluorescent, conjugated antibodies, enabling the examination of dot-like structures called puncta, which indicate intact lysosomes. The loss of these puncta, along with fluorescent cytosolic dispersion, indicates lysosomal membrane permeabilization (LMP) and intracellular cytosolic translocation of lysosomal contents. The loss of puncta and cytosolic dispersion was observed following exposure to rigid carbon (nano)fibers (Sun et al. 2015; Svadlakova et al. 2020), but not exclusively, as it has been caused by entangled carbon NFs (Cohignac et al. 2018; Sun et al. 2015) and HARNs (Cohignac et al. 2018; Dong et al. 2013; He et al. 2018; Sohaebuddin et al. 2010; Yang et al. 2014). In contrast, this phenomenon has not been observed for non-fibrous carbon materials (Cohignac et al. 2018; He et al. 2018; Sun et al. 2015; Svadlakova et al. 2020).

However, these results do not address the question of whether LMP happens due to cellular death. When comparing experiments that assess LMP and intracellular protein translocation with those using cell membrane permeabilization as a readout for cytotoxicity, positive results for LMP were observed in the absence of cytotoxicity for rigid carbon (nano)fibers (Svadlakova et al. 2020), entangled carbon NFs (Cohignac et al. 2018) and HARNs (Cohignac et al. 2018; Dong et al. 2013). However, these events were also observed in parallel with cytotoxicity for HARNs (He et al. 2018; Yang et al. 2014). For non-fibrous materials, no LMP has been detected, although cytotoxicity was observed (Cohignac et al. 2018), while other studies indicated negative results for both LMP and cytotoxicity (He et al. 2018; Svadlakova et al. 2020). Collectively, these findings suggest a structure-activity relationship, in which all morphologies of carbon-NMs, except non-fibrous ones, cause LMP and lysosomal content leakage as a proceeding step to cytotoxicity.

#### Colorimetric assays

In six out of the 62 studies, the neutral red uptake (NRU) assay was employed to assess LMP. Neutral red is a cationic dye that selectively accumulates in intact lysosomes, but leaks out as a result of LMP (Repetto et al. 2008). Rigid carbon (nano)fibers caused a loss of lysosomal dye retention (Cheng et al. 2009; Di Cristo et al. 2019; Ledwith et al. 2025), while no significant neutral red loss was observed for HARNs (Di Cristo et al. 2019; Ledwith et al. 2025; Long et al. 2019) or non-fibrous carbon NMs (Ledwith et al. 2025). None of these studies showed NRU results for entangled carbon NFs. Although fewer studies applied the NRU assay compared to those employing fluorescence microscopy to evaluate lysosomal leakage, the available evidence still suggests a structure-activity relationship linking rigid carbon (nano)fibers with LMP.

#### Immunoassays for NLRP3 inflammasome activation

Of the 62 studies reviewed, 21 specifically investigated NLRP3-inflammasome associated release of the cytokine IL-1β upon macrophage exposure to rigid carbon (nano)fibers, as well as to another morphology of carbon-based NMs as a control counterpart. Among these, eight studies reported extracellular release of IL-1β exclusively by rigid carbon (nano)fibers, with no release observed for other evaluated material morphologies (Busch et al. 2022; Hamilton et al. Jr 2018; Lim et al. 2025; Murphy et al. 2012; Omori et al. 2021; Palomäki et al. 2015; Palomaki et al. 2011; Svadlakova et al. 2020). In contrast, seven studies show that both rigid carbon (nano)fibers, as well as entangled carbon NFs, led to IL-1β release (Cui et al. 2016; Fujita et al. 2020; Hindman and Ma 2019; Keshavan et al. 2021; Lee et al. 2018; Murphy et al. 2022; Sun et al. 2015). However, among these seven studies, the IL-1β release was most pronounced for rigid carbon (nano)fibers in all but two studies, which reported comparable responses for entangled carbon NFs (Hindman and Ma 2019; Sun et al. 2015). Two studies also reported IL-1β release by both rigid carbon (nano)fibers and particulate carbon black, although higher levels of the cytokine were released by the rigid carbon (nano)fibers (Brown et al. 2010; Haniu et al. 2011). Conversely, four studies found no IL-1β release upon exposure to either rigid carbon (nano)fibers or any other material morphology (Bhattacharya et al. 2017; Boyles et al. 2015; Chortarea et al. 2019; Sweeney et al. 2015). In one of these studies, Mitsui-7 did not induce IL-1β release, but it was observed via Cryo-EM to cause frustrated phagocytosis (Boyles et al. 2015). Moreover, another study reported that Mitsui-7 did not induce IL-1β release, while entangled carbon NFs and shorter HARNs did (Hautanen et al. 2023).

Among the 62 studies, only two measured IL-18 release. One reported its release by Mitsui-7 and other rigid carbon (nano)fibers, but there were no other carbon-based NMs included for comparison (Fujita et al. 2020). The other study found no IL-18 release upon exposure to rigid carbon (nano)fibers, nor carbon black (Sanchez et al. 2011). Among the studies, when cell membrane permeabilization was also measured as a readout for cytotoxicity, NLRP3 inflammasome activation (indicated by either IL-1β or IL-18 release) upon response to rigid carbon (nano)fibers, occurred in the absence of cytotoxicity (Palomäki et al. 2015; Svadlakova et al. 2020), occurred before the onset of cytotoxicity (Lim et al. 2025; Murphy et al. 2012), or in parallel to cytotoxicity (Busch et al. 2022; Haniu et al. 2011; Keshavan et al. 2021). Additionally, one study showed NLRP3 inflammasome activation only occurred in macrophages exposed to rigid carbon (nano)fibers at concentrations above those that induced metabolic disruption (Fujita et al. 2020), while others reported no NLRP3 activation but observed metabolic disruption (Bhattacharya et al. 2017; Hautanen et al. 2023; Sweeney et al. 2015).

Collectively, these findings suggest a structure-activity relationship for NLRP3 inflammasome activation, where rigid carbon (nano)fibers tend to induce higher degrees of activation and cytokine release, compared to other carbon-based NM morphologies. Furthermore, for rigid carbon (nano)fibers, inflammasome activation typically precedes cell membrane permeabilization, but occurs after disruption of metabolic activity. However, overall, the NLRP3 inflammasome activation resulting from the intracellular translocation of lysosomal proteins is not an exclusive response to rigid carbon-fibers. Therefore, measuring NLRP3 inflammasome activation may not be a reliable in vitro marker for predicting fiber-like pathogenicity for NFs.

### Extracellular lysosomal proteins translocation

The translocation of lysosomal proteins into the extracellular environment has been observed in macrophages exposed to various NFs (Stobernack et al. 2026) as well as filamentous pathogens (Maxson et al. 2018; Prashar et al. 2013), suggesting a key role for morphology-driven responses. The significance of these findings lies in the fact that the release of lysosomal content into the extracellular space is considered a hallmark of frustrated phagocytosis. However, since the release of lysosomal proteins may also occur as a result of cell death, measuring the release of these proteins in parallel with the release of other cytosolic proteins could help clarify if the release of these lysosomal proteins is occurring independently of cell death, therefore inferring a mechanism associated with frustrated phagocytosis. A commonly used assay to measure cell membrane permeabilization is the extracellular release of the cytosolic lactate dehydrogenase (LDH).

In eight out of the 62 studies, the extracellular lysosomal content release was assessed. Mitsui-7 was shown to cause the extracellular release of cathepsin B (Keshavan et al. 2021; Lim et al. 2023; Palomäki et al. 2015; Palomaki et al. 2011), as well as other cathepsins and lysosomal luminal proteins (Stobernack et al. 2026). Notably, in two of these studies, a six-hour exposure resulted in no LDH release (Palomäki et al. 2015; Palomaki et al. 2011), while other studies show LDH release after 18–24 h of exposure, but only at significantly higher concentrations than those required for the extracellular release of lysosomal luminal proteins (Lim et al. 2025; Stobernack et al. 2025). Another study reported the significant release of both lysosomal luminal proteins and LDH at the same exposure concentrations following 24-hour exposure (Keshavan et al. 2021). On the other hand, for entangled carbon NFs, studies have reported no extracellular release of lysosomal luminal proteins nor LDH (Palomäki et al. 2015; Palomaki et al. 2011). In contrast, others have reported the release of several cathepsins and lysosomal luminal proteins by entangled carbon NFs (Fletcher et al. 2021; Jessop et al. 2017), and cathepsin B by HARNs (Bussy et al. 2013). However, in these studies with carbon NFs, since no concurrent LDH assessment was conducted, it cannot be concluded whether these responses occurred independently of cytotoxicity. For non-fibrous carbon NMs, no extracellular release of lysosomal luminal proteins was observed at exposure times ≤ 24 h (Lim et al. 2023; Stobernack et al. 2026). 

Altogether, it appears that the release of lysosomal proteins into the extracellular environment occurs for rigid carbon (nano)fibers, and to a lesser extent, by entangled carbon NFs and HARNs. For rigid carbon (nano)fibers, this phenomenon seems to precede cell membrane permeabilization. Altogether, in the case of rigid carbon (nano)fibers, it appears that both the intracellular and extracellular release of lysosomal proteins occurs. Below, we examine how rigid carbon (nano)fibers interact with lysosomes and the fate of lysosomal proteins during morphology-driven cellular responses.

## Rigid carbon (nano)fibers lead to the intracellular lysosomal luminal protein translocation

Macrophages internalize targets primarily via phagocytosis. In the canonical phagocytic pathway, actin-driven pseudopods surround and engulf the targets, and, following pseudopod fusion, a phagosome is formed that subsequently undergoes scission from the plasma membrane (Donaldson et al. 1992). The phagosome containing the sequestered targets undergoes maturation by fusing with lysosomes to form phagolysosomes, which subsequently become enriched with lysosomal hydrolases, such as cathepsins, creating an acidic and degradative milieu (Maxson et al. 2018; Nguyen and Yates 2021). However, this compartmentalization can make lysosomes vulnerable to damage, potentially compromising their structural integrity (Stern et al. 2012). Such damage may arise from chemically driven mechanisms, such as oxidative stress or osmotic swelling, as well as from mechanical forces, potentially via morphology-driven mechanisms (Zhang et al. 2025). 

Although phagocytic vesicles are highly dynamic and can enlarge to accommodate internalized materials, this enlargement can exceed the typical resting lysosome size of 0.1 to 1 μm, increasing membrane tension. Such tension has been hypothesized to potentially cause mechanical rupture (Keshavan et al. 2021; Scott et al. 2025). However, the exact mechanism remains a matter of research. Several studies addressing NF uptake postulated that physical shearing by needle-like NMs is the cause of observed lysosomal rupture. However, these studies have not conclusively proven this hypothesis (Bunderson-Schelvan et al. 2017; Keshavan et al. 2021). For instance, Zhu et al. (2016) used a theoretical coarse-grain molecular analysis of Mitsui-7 compartmentalized within a lysosome, finding that membrane tension-induced rupture is unlikely to be the primary cause for lysosomal damage, but most likely, the sustained forced contact between the tip of the NF and the inner leaflet of the lysosomal membrane is responsible for phospholipid extraction and, later, lysosomal rupture. This can be explained due to MWCNTs composition of concentric tubes of graphite, which consist of rings with sp² hybridized carbon atoms that form a delocalized π (pi) electron cloud. The nonpolar, hydrophobic surface of pristine MWCNTs may preferentially interact with other nonpolar molecules, such as the lipid tails. The lipid bilayer of the lysosomal membrane is primarily composed of phospholipids, along with cholesterol, glycolipids, and specialized membrane proteins. This phospholipid extraction has been observed in several studies using coarse-grained molecular dynamics simulations with graphene oxide (Duan et al. 2015). Consequently, phospholipid extraction reduces the phospholipid density within the lysosomal membrane, weakening the connection between the phospholipids, which ultimately leads to LMP and the intracellular translocation of luminal proteins. Studies have shown that dispersing rigid carbon NFs in an F108 polymer, which forms an acid-stable 'brush-like' layer providing steric hindrance, significantly reduces lysosomal damage by preventing phospholipid extraction. In contrast, the protein corona formed by dispersing the NFs in bovine serum albumin media offers only temporary protection, as it eventually is degraded by lysosomal hydrolases, exposing the bare material (Wang et al. 2011, 2013).

Unlike rigid NFs, entangled carbon NFs and non-fibrous NMs are not mechanically forced into prolonged contact with lysosomal membranes due to geometrical constraints (Zhu et al. 2016). It is also important to note that the carbon NFs must be sufficiently rigid to resist additional buckling under lysosomal compressive forces (~ 20 pN) (Zhu et al. 2016). A key study by Sohaebuddin et al. (2010) using three HARNs of equal length (2 μm) but different diameters, showed that only the rigid material (diameter > 50 nm), but not the non-rigid HARNs (diameter < 20 nm), caused lysosomal damage and LMP. This suggests that sufficiently rigid NMs with an elongated morphology, even if shorter than a critical fiber, may cause lysosomal damage if exceeding the postulated lysosomal length threshold of 1 μm. This requirement for both sufficient rigidity and length to potentiate lysosomal damage may explain why rigid carbon (nano)fibers often cause greater intracellular translocation of lysosomal luminal proteins and subsequent NLRP3 inflammasome activation, even though these cellular responses are not exclusive to rigid carbon (nano)fibers. In addition to carbon-based NFs, several in vitro macrophage studies assessing toxicity of NFs of various chemical compositions, along with corresponding TEM analysis which indicates these NFs appear rigid, detected lysosomal damage and intracellular luminal protein translocation. These cellular events are observed for NFs composed of cerium dioxide (Ji et al. 2012; Lin et al. 2014), titanium dioxide (Hamilton et al. 2009, 2014; Jessop et al. 2017; Stobernack et al. 2025), nickel (Poland et al. 2012), and silicon carbide (Bruch et al. 2014; Stobernack et al. 2025), but not for their particulate counterparts.

### The intracellular translocation of cathepsins can activate the NLRP3 inflammasome and cell death cascades

As a result of phospholipid extraction-induced LMP, multiple lysosomal proteins are translocated into the cytosol (Muñoz-López et al. 2020; Zhu et al. 2016). Notably, although lysosomal cathepsins typically require an acidic pH for optimal proteolytic activity, they may undergo structural adaptations and substrate binding that allow them to sustain their functionality in more alkaline, cytosolic conditions. Furthermore, LMP may result in cytosolic acidification (Villamil Giraldo et al. 2014). In terms of NLRP3 inflammasome activation, while many studies suggest that cathepsin B is the primary enzyme responsible, other cathepsins may also play a role (Campden and Zhang 2019). In addition, several in vitro studies have shown that cathepsin B inhibition alone is insufficient to reduce IL-1β secretion (Hamilton et al. 2014; Lim et al. 2025; Palomaki et al. 2011; Wang et al. 2011). Furthermore, the cytosolic presence of cathepsins may contribute to plasma membrane permeabilization and subsequent cell death. Partial release of cathepsins from lysosomes represents a critical step in lysosome-mediated apoptosis, whereas extensive lysosomal rupture and the massive release of cathepsins can lead to the degradation of essential cellular proteins, ultimately resulting in autolysis and necrotic cell death (Boya and Kroemer 2008).

In vivo activation of NLRP3 inflammation and consequent IL-1β release may contribute to the pathogenesis of inflammatory diseases. It is known that IL-1β release from macrophages mediates inflammation-induced tissue injury that can progress to fibrosis and tumorigenesis (Murphy et al. 2021; Sayan and Mossman 2016; Wang et al. 2017). Additionally, IL-1β release is implicated in various inflammation-related diseases, including arthritis, prion disease, and Alzheimer’s (Chen et al. 2023; Kelley et al. 2019; Yang et al. 2019). In the context of fibrosis, macrophage-derived IL-1β promotes TGF-β production by fibroblasts and can directly induce their transformation into myofibroblasts. This process enhances extracellular matrix (ECM) protein secretion, such as collagen, contributing to tissue thickening and fibrosis (Liu et al. 2024; Yang et al. 2023). In the development of MPM, inflammation-related signaling between IL-1β derived from tumor-associated macrophages and the interleukin-1 receptor on mesothelial cells contributes to their acquisition of a cancer stem cell-like phenotype (Horio et al. 2020). Notably, the IL-1β receptor antagonist anakinra has been trialed as a therapeutic agent for MPM, showing a marked delay in the median time of MPM onset and prolonged survival (Kadariya et al. 2016, 2022). Several in vivo studies, summarized in Supplementary Table III, compare pathological outcomes in NLRP3 inflammasome-deficient (NLRP3^–/–^) mice and wild-type (WT) mice when exposed to asbestos or Mitsui-7. These studies show that NLRP3^–/–^ mice have reduced IL-1β levels in lung fluids and exhibit attenuated pathological outcomes, including pulmonary fibrosis, granuloma formation, peritoneal wall thickening, and MPM (Arnoldussen et al. 2018; Chow et al. 2012; Dostert et al. 2008; Kadariya et al. 2016; Poland et al. 2008; Rydman et al. 2015; Thompson et al. 2017). However, although NLRP3^–/–^ mice show reduced MPM incidence and delayed development, MPM is not completely avoided (Chow et al. 2012; Kadariya et al. 2016).

Although LMP, intracellular lysosomal luminal protein translocation, NLRP3 inflammasome activation, and IL-1β release occur in response to pathogenic fiber exposure, these events are not exclusive to pathogenic fibers. As such, in vitro evaluation of the relocation of these lysosomal proteins may not reliably indicate frustrated phagocytosis. This is evidenced by various in vivo and epidemiological studies on crystalline silica exposure, which demonstrate that this substance also has a high capacity to cause lysosomal damage, NLRP3 inflammasome activation, and IL-1β secretion, ultimately leading to a fibrosing lung disease (silicosis) and increasing the risk of lung cancer (Cassel et al. 2008; Hornung et al. 2008; Leinardi et al. 2020; Sato et al. 2018). However, due to the in vivo physiological clearance mechanisms, crystalline silica is not retained in the lungs; thus, these pathologies are typically associated with chronic inflammation caused by repeated exposure, such as in occupational settings. In contrast, chronic inflammation caused by long pathogenic fibers like asbestos arises from frustrated phagocytosis, as these excessively long fibers are retained in the lungs and can persist in vivo indefinitely, potentially even after a single exposure. This may explain why, unlike asbestosis, clinical cases of silicosis are not associated with MPM malignancies (deShazo 1982). Altogether, the intracellular translocation of lysosomal luminal proteins and NLRP3 inflammasome activation is a mechanistic response related to the inhalation of pathogenic fibers, but may be better suited as general indicators of potential in vivo inflammation, and not necessarily associated with frustrated phagocytosis.

## Rigid carbon (nano)fibers capable of frustrated phagocytosis cause lysosomal content release into the extracellular environment

As previously described in the canonical pathway for macrophage phagocytosis, lysosomes migrate to and fuse with nascent phagosomes to form phagolysosomes. However, during frustrated phagocytosis, pseudopods are unable to contort to excessive lengths imposed by critically long fibers such as Mitsui-7 and asbestos, resulting in the formation of an open phagocytic cup, also referred to as a frustrated phagosome (Champion and Mitragotri 2006; Mularski et al. 2018; Uribe-Querol and Rosales 2020). In this scenario, lysosomes fuse with these open phagocytic cups, resulting in lysosomal contents being released into the ECM (Stobernack et al. 2026). In contrast, lysosomal membrane proteins like LAMP1 are retained in the membrane, forming the phagocytic cup (Brahmendra 2015; Eng et al. 2007; Labrousse et al. 2011; Maxson et al. 2018; Prashar et al. 2013; Stobernack et al. 2025, 2026). Studies using fluorescent lysosomal probes and LAMP1-conjugated antibodies have visualized this accumulation of LAMP-1 in the phagocytic cup in vitro, following macrophage exposure to crocidolite asbestos or to non-phagocytosable immune complex-coated surfaces (Brahmendra 2015; Kovari et al. 2016; Labrousse et al. 2011; Mularski et al. 2018; Zurier et al. 1973). These cellular events are expected to occur soon after the recognition and attempted engulfment of the fiber, and therefore, the extracellular release of the lysosomal luminal proteins should be observed as an early event during frustrated phagocytosis.

In this context, it is interesting to review the interplay between asbestos and the selective release of lysosomal enzymes. To this end, Supplementary Table IV shows that various forms of asbestos, including crocidolite (Driscoll et al. 1990; Wright et al. 1983), chrysotile (Davies et al. 1974; Dean et al. 1979; Kang et al. 1979; Lock et al. 1987; Morgan et al. 1977; Schorlemmer et al. 1977), and amphibole (Jaurand et al. 1980), employed for in vitro exposure of macrophages, result in the selective extracellular release of lysosomal luminal proteins, without a concurrent release of LDH, which is a cytosolic enzyme easily released when the cell membrane starts becoming permeable. This is comparable to in vitro studies of Mitsui-7 exposure for six-hours, which resulted in no LDH release, but the release of several cathepsins (Palomäki et al. 2015; Palomaki et al. 2011). However, this pattern of selective extracellular release of lysosomal luminal protein is not always consistent for all different types of asbestos fibers, which may be attributed to the heterogeneity in fiber dimensions and chemical compositions, which cause distinct cellular responses. Other studies, as shown in Supplementary Table IV, report a time-dependent release of both LDH and lysosomal luminal enzymes (Davies 1980; Jaurand et al. 1981; Li et al. 2012; Pailes et al. 1984; Pietrofesa et al. 2016). Fundamentally, many of these studies employ time-course analysis, showing that lysosomal luminal proteins are generally released within the first 6 to 12 h post-exposure, whereas the release of LDH tends to increase only after 12 h (Beck et al. 1971; Bruch 1974; Jaurand et al. 1981; Li et al. 2012; Pailes et al. 1984). These findings support the postulated mechanism that the lysosomal luminal proteins are released into open phagocytic cups as an early cellular mechanism due to frustrated phagocytosis, prior to cell membrane permeabilization. Notably, in vivo studies support these results: exposure to various asbestos species is associated with increased levels of multiple lysosomal proteins, including cathepsins, as well as LDH, in bronchoalveolar lavage fluid and pleural lavage fluid (Adamson and Bowden 1986; Edwards et al. 1984; Jajte et al. 1987; Leonard et al. 2002; Sjöstrand et al. 1989; Viswanathan et al. 1973). Additionally, multiple cathepsins are upregulated at both mRNA and protein levels in lung tissue after long latency periods post exposure (Sabo-Attwood et al. 2005). Similar findings are observed in vivo following exposure to Mitsui-7 (Hamilton et al. Jr 2018; Sager et al. 2014).

The above-described phenomena are not exclusively of fibrous materials, as other examples of frustrated phagocytosis are observed in nature. When macrophages encounter various pathogens with filamentous morphology and excessive lengths, such as filamentous *Legionella pneumophila* (Prashar et al. 2013) and *Candida albicans hyphae* (Maxson et al. 2018), long-lasting open tubular phagocytic cups are formed. These cups fail to develop hydrolytic capacity, and the extracellular release of fluorescent lysosomal probes and cathepsins is observed in the absence of cell membrane permeabilization (Maxson et al. 2018; Prashar et al. 2013). This allows the pathogens to evade the immune system. Moreover, a comparable pattern of selective extracellular lysosomal protein release is observed when macrophages encounter non-filamentous physiological targets that cannot be phagocytosed, either due to their excessive size or because they are embedded in a dense ECM. A mechanism referred to as digestive exophagy may occur when the macrophage attempts to extracellularly catabolize these unphagocytosable targets. For example, this pattern has been observed for macrophages exposed in vitro to dead adipocytes, which can be a hundred times larger than macrophages (Haka et al. 2016), as well as in response to large lipoprotein aggregates, such as those tightly cross-linked to the subendothelial matrix in atherosclerotic plaques (Haka et al. 2009). Notably, clinical samples of coronary atherosclerotic lesions are shown to be positively stained for various lysosomal luminal proteins, including cathepsins (Hakala et al. 2003; Marathe et al. 1999). Similarly, in vitro studies with microglia, the resident macrophages of the brain, exposed to large deposits of fibrillar amyloid-β plaques associated with Alzheimer’s disease, have demonstrated the extracellular release of fluorescent lysosomal probes from viable macrophages (Jacquet et al. 2024). These plaques in in vivo Alzheimer brain models also stain positively for multiple cathepsins (Cataldo et al. 1990). Collectively, the results from studies on pathogenic fibers, filamentous pathogens, and excessively large pathogens apparently support a morphologically-driven mechanism for selective lysosomal enzyme release from in vitro macrophages.

Due to the failure of the immune cells to clear targets of critical morphology, it is to be expected that a chronic release and increased levels of lysosomal luminal proteins, particularly cathepsins, occurs in the surrounding tissues, as a result of macrophage frustrated phagocytosis. This may be linked to the development of the pathogenicity of various chronic inflammatory diseases and cancer. Specifically related to fiber toxicity, increased in vivo levels of cathepsins in lung fluids and tissue are associated with lung fibrosis (Brain 1980; Davis 1967). Due to their proteolytic activity, cathepsins can degrade ECM components, which in turn activate pro-fibrotic factors (Chen et al. 2025; van den Brûle et al. 2005; Yoo et al. 2022). The degradation of ECM components, including collagen, fibronectin, proteoglycans, elastin, and basement membranes, can also facilitate cancer cell migration and tumor invasion, thereby advancing malignancy (Olson and Joyce 2015). Moreover, cathepsins are upregulated in lung cancer and MPM (Hosako et al. 2012; Kayser et al. 2003; Wali et al. 2009; Zhou et al. 2012). In several cancers, the increase of cathepsins levels is proposed as a potential biomarker of disease progression (Mohamed and Sloane 2006; Nomura and Katunuma 2005; Yadati et al. 2020). Collectively, the extracellular release of lysosomal luminal proteins in the absence of cytotoxicity in vitro may be early evidence of frustrated phagocytosis. This may be a morphologically driven mechanism, caused by long, filamentous, or fibrous targets which impede the closure of the phagocytic cup, or due to the macrophage purposefully expelling its lysosomal content in an attempt to extracellularly digest excessively large targets. In cases where targets are phagocytosed but phagocytosis is aborted, the macrophage may also expel its contents in an attempt to expel indigestible targets. Several in vivo studies have linked the extracellular release of lysosomal luminal proteins, such as cathepsins, to inflammatory diseases and cancer, supporting the idea that this mechanism plays a role in pathogenesis.

## Integration into a unified mechanistic model

**Fig. 2:**
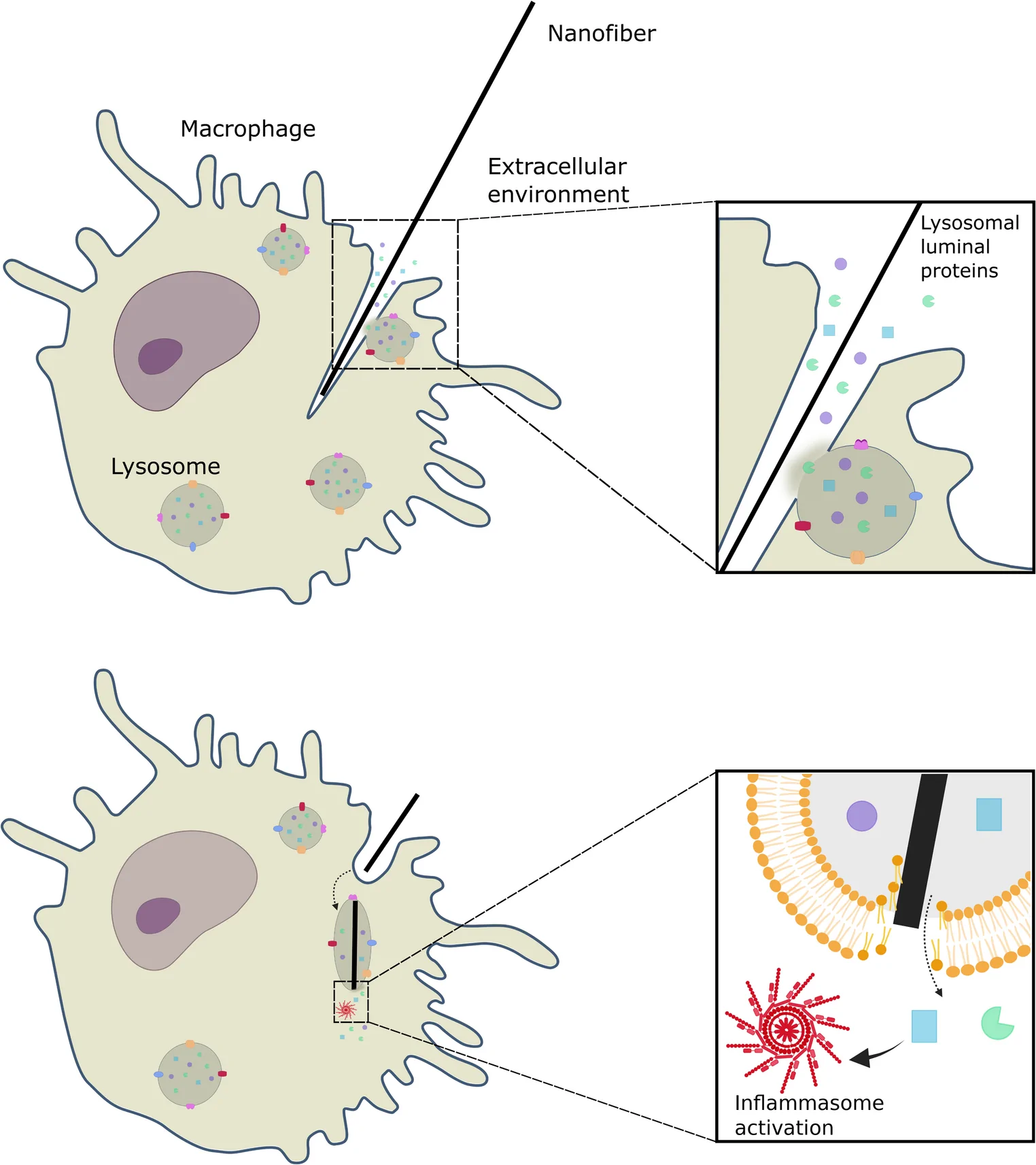
(Top) Release of lysosomal luminal contents into the extracellular space during frustrated phagocytosis, resulting from the fusion of a lysosome with an open phagocytic cup. (Bottom) Release of lysosomal luminal contents into the cytosol due to lysosomal membrane permeabilization, which occurs as a consequence of phospholipid extraction from lysosomal membrane proteins following sustained tip contact between the (nano)fiber and the inner leaflet of the lysosomal membrane. Adapted from Stobernack et al.(2026), licensed under CC BY 4.0. Created in BioRender.com Ribalta, C. (2026). https://BioRender.com/w8ocg0b

Focusing on the reviewed studies and aiming to integrate the experimental findings, Fig. 2 shows a proposed unified model of fiber toxicity. This model accounts for fiber length heterogeneity, reconciling observations that rigid carbon fibers induce both intra- and extracellular translocation of lysosomal proteins. These effects may be explained by the broad size distribution of fibers Mitsui-7, which contain both short and long fiber fractions (Chortarea et al. 2019). Shorter fibers primarily induce LMP, accompanied by activation of the NLRP3 inflammasome (Fig. 2, bottom), whereas longer fibers induce frustrated phagocytosis (Fig. 2, top). The model also addresses the question of how lysosomal proteins are released extracellularly, suggesting that this process represents a distinct mechanism associated with frustrated phagocytosis, and arising from lysosomal fusion with an open phagocytic cup. Both of these mechanisms can occur simultaneously and likely before the cathepsins in the cytosol are able to initiate cell death cascades.

## Applicability for an in vitro testing strategy

Using robust and relevant cell models, along with harmonized cell culture conditions, is a fundamental prerequisite for establishing an in vitro testing strategy for critical NFs, as variations in these conditions can significantly influence experimental outcomes (Ledwith et al. 2024). Previous in vitro testing strategies have focused on screening assays that measure cytotoxicity, oxidative stress, and inflammation, which represent key cellular responses to critical fibers (Ledwith et al. 2025). Building on these approaches and the proposed model for fiber toxicity, whereby lysosomes fuse with open phagocytic cups during frustrated phagocytosis, we suggest that the extracellular release of lysosomal proteins, in the absence of cell membrane permeabilization, may serve as an in vitro biological marker for predicting fiber-like pathogenicity of NFs. To accurately interpret this phenomenon, concurrent measurements of the extracellular cytosolic proteins, such as LDH, are necessary to determine whether extracellular lysosomal protein release is a morphology-driven mechanism associated with frustrated phagocytosis, independent of, or prior to, cell death. Correspondingly, the levels of these proteins in cell lysates should be measured at shorter exposure times, which may be more suitable for characterizing the selective release of lysosomal luminal enzymes before significant cytotoxicity occurs. In contrast, longer exposures, such as 24 h, may obscure this cellular response, as cytotoxicity resulting from intracellular lysosomal damage, or other mechanisms, including oxidative stress, may already be present.

## Summary and outlook

This review examined the effects of exposing macrophages to various carbon-based NMs of different morphologies, including MWCNTs and HARNs, with the aim of determining whether lysosomal damage and the translocation of lysosomal luminal enzymes, both intracellularly and extracellularly, represent cellular responses to pathogenic NFs and indicators of macrophage frustrated phagocytosis. In light of gathered evidence, a morphology-driven mechanism is proposed that involves two concurrent pathways accounting for fiber length heterogeneity. First, rigid carbon (nano)fibers belonging to the shorter fraction, small enough to be phagocytosed by macrophages, may cause geometric constraints when confined within lysosomal vesicles. This confinement may promote phospholipid extraction from the lysosomal membrane, leading to lysosomal membrane permeabilization and subsequent translocation of lysosomal luminal proteins into the cytosol. Among these proteins, cathepsin B can activate the NLRP3 inflammasome, triggering the release of inflammatory cytokines into the extracellular space. However, lysosomal damage and NLRP3 inflammasome activation are not exclusive indicators of frustrated phagocytosis in response to pathogenic long fibers. Second, the longer fraction of rigid carbon fibers, which exceeds the macrophage’s capacity for engulfment, can induce frustrated phagocytosis by delaying or preventing closure of the phagocytic cup. Fusion of lysosomes with the open phagocytic cup leads to the extracellular release of lysosomal luminal proteins, an early cellular response that occurs before cell membrane permeabilization. This phenomenon has also been observed with other long, filamentous bacteria and large pathogens found in nature that trigger frustrated phagocytosis. Various in vivo and clinical studies have suggested that the extracellular release of lysosomal luminal proteins, particularly cathepsins, may be fundamental to the pathogenesis of inflammatory diseases and fiber toxicity-related conditions, such as lung fibrosis, lung cancer, and MPM. Along with pro-inflammatory mediators and reactive oxygen species, the release of cathepsins by activated macrophages during chronic inflammation may represent an overlooked cellular KE in fiber-related pathologies and could be incorporated into relevant AOPs.

## Supplementary Information

Below is the link to the electronic supplementary material.

Supplementary Material 1

Supplementary Material 2

Supplementary Material 3

Supplementary Material 4

Supplementary Material 5

## Data Availability

No datasets were generated or analysed during the current study.

## Abbreviations

AOPs: Adverse outcome pathways
ECM: Extracellular matrix
HARN: High-aspect ratio nanomaterials
KE: Key event
LMP: Lysosomal membrane permeabilization
MIE: Molecular initiating event
MoA: Mode of action
MPM: Malignant pleural mesothelioma
MWCNTs: Multi-walled carbon nanotubes
NF: Nanofiber
NM: Nanomaterial
NRU: Neutral red uptake
WHO: World Health Organization
