# Supplementary material for: Involvement of lysosomal proteins in morphology-driven toxicity of (nano)fibers
Source: Arch Toxicol. 2026 Jul 14;100(12):5349–64. doi: 10.1007/s00204-026-04503-y (PMC13748141; doi:10.1007/s00204-026-04503-y)
Supplement: Supplementary file 1 [file 204_2026_4503_MOESM5_ESM.docx]

# **Supplementary Material**

**Involvement of Lysosomal Proteins in Morphology-Driven Toxicity of (Nano)fibers**

Rico Ledwith, Carla Ribalta, Mario Pink, Andrea Haase and Verónica I. Dumit^*^

R. Ledwith, C. Ribalta, M. Pink, A. Haase and V.I. Dumit

German Federal Institute for Risk Assessment (BfR), Department of Chemical and Product Safety, Berlin, Germany

R. Ledwith and A. Haase

Freie Universität Berlin, Institute of Pharmacy, Berlin, Germany

A. Haase

University of the Western Cape (UWC), Department of Chemical Sciences, Bellville 7535, South Africa

C. Ribalta

Federal Institute for Occupational Safety and Health (BAuA), Unit of Materials and Particulate Hazardous Substances, Nöldnerstraße 40-42 Berlin, Germany

*Corresponding author e-mail: [Veronica.Dumit@bfr.bund.de](mailto:Veronica.Dumit@bfr.bund.de)

ORCID: 0009-0002-5091-4284

**Supplementary Information:**

Supplementary Table I: Paper search strategy and terms

Supplementary Table II: *In vitro* macrophage studies involving exposure to different morphologies of carbon-based nanomaterials

Supplementary Table III: *In vivo* studies employing knockout mice and asbestos or Mitsui-7 exposure

Supplementary Table IV: *In vitro* macrophage studies involving exposure to crystalline silica or asbestos
